# Supplementary material for: Identification of fallopian tube microbiota and its association with ovarian cancer
Source: eLife. 2024 Mar 7;12:RP89830. doi: 10.7554/eLife.89830 (PMC10942644; doi:10.7554/eLife.89830)
Supplement: Supplementary file 1. [file elife-89830-supp1.docx]

**Supplemental Table 1.** Summary of samples sequenced.

| **Sample Type** | **Total Samples** | **Ovarian cancer** | | **Non-cancer** | |
| --- | --- | --- | --- | --- | --- |
|  |  | **Samples** | **Patients** | **Samples** | **Patients** |
| **No Template PCR Control – Water** | 111 | N/A | N/A | N/A | N/A |
| **DNA Extraction Control – Buffer** | 36 | N/A | N/A | N/A | N/A |
| **Operating Room Air** | 130 | 53 | 53 | 77 | 77 |
| **Laparoscopic Port** | 81 | 8 | 4 | 73 | 45 |
| **Paracolic Gutter** | 122 | 73 | 70 | 49 | 48 |
| **Fallopian Tube and Ovarian Surface** | 369 | 160 | 72 | 209 | 99 |
| **Cervix** | 152 | 65 | 65 | 87 | 87 |
| **TOTAL** | 1001 | 359 | 81 | 495 | 106 |
