# Supplementary material for: Identification of fallopian tube microbiota and its association with ovarian cancer
Source: eLife. 2024 Mar 7;12:RP89830. doi: 10.7554/eLife.89830 (PMC10942644; doi:10.7554/eLife.89830)
Supplement: Supplementary file 2. [file elife-89830-supp2.docx]

**Supplemental Table 2.** Bacterial concentration (log10[16S rRNA genes/μl of DNA]) of each sample type and the p-value of each comparison.

| **Sample type** | **Mean** | **Standard Deviation** | **P-value**  **(compared to FT samples)** |
| --- | --- | --- | --- |
| **Cervix** | 4.912207 | 1.553575 | <0.001 |
| **Fallopian tube** | 0.3975917 | 0.6684811 | N/A |
| **Paracolic gutter** | 0.2764742 | 0.7500778 | 0.11 |
| **Laparoscopic port** | -0.3592626 | 0.6236524 | <0.001 |
| **Air** | -0.8201336 | 0.6245525 | <0.001 |
| **Buffer** | -1.044277 | 0.5530808 | <0.001 |
