# Supplementary material for: Identification of fallopian tube microbiota and its association with ovarian cancer
Source: eLife. 2024 Mar 7;12:RP89830. doi: 10.7554/eLife.89830 (PMC10942644; doi:10.7554/eLife.89830)
Supplement: Supplementary file 3. [file elife-89830-supp3.docx]

**Supplemental Table 3.** Processing steps and result summary after each step.

|  | **Processing Summary** | **Samples and taxa after each step** |
| --- | --- | --- |
| 1 | Starting set | 1001 Samples |
| 2 | Merged Sample by Participant and Sample Type | 767 Samples X 1337 Taxa |
| 3 | Filter #1. Remove Taxa present in only 1 Sample | 767 Samples X 892 Taxa |
| 4 | Filter #2. Remove Taxa present in > 1 No Template controls | 645 Samples X 881 Taxa |
| 5 | Filter #3. Remove Taxa present in DNA extraction controls | 619 Samples X 836 Taxa |
| 6 | Filter #4. Remove Taxa in > 1 AIR samples | 575 Samples X 753 Taxa |
| 7 | Remove No Template Controls, DNA Extraction Controls, and AIR Controls | 479 Samples X 753 Taxa |
| 8 | Filter #5. Filter Taxa for presence in at least 1 Fallopian Tube and Ovarian surface (FTO) sample | 457 Samples X 715 Taxa |
| 9 | Filter #6. Filter Taxa for presence in at least 1 cervical sample | 457 Samples X 84 Taxa |
| 10 | Filter #7. Filter for final sample set. At least 1 read for a given sample | 340 Samples X 84 Taxa |
