# Supplementary material for: Identification of fallopian tube microbiota and its association with ovarian cancer
Source: eLife. 2024 Mar 7;12:RP89830. doi: 10.7554/eLife.89830 (PMC10942644; doi:10.7554/eLife.89830)
Supplement: Supplementary file 4. — Each number is the percentage of individuals in each category with the presence of each bacterial species. [file elife-89830-supp4.docx]

**Supplemental Table 4.** Comparison of overall and laparotomy cases in the bacterial prevalence in FT samples from ovarian cancer versus non-cancer patients. Each number is the percentage of individuals in each category with the presence of each bacterial species.

**
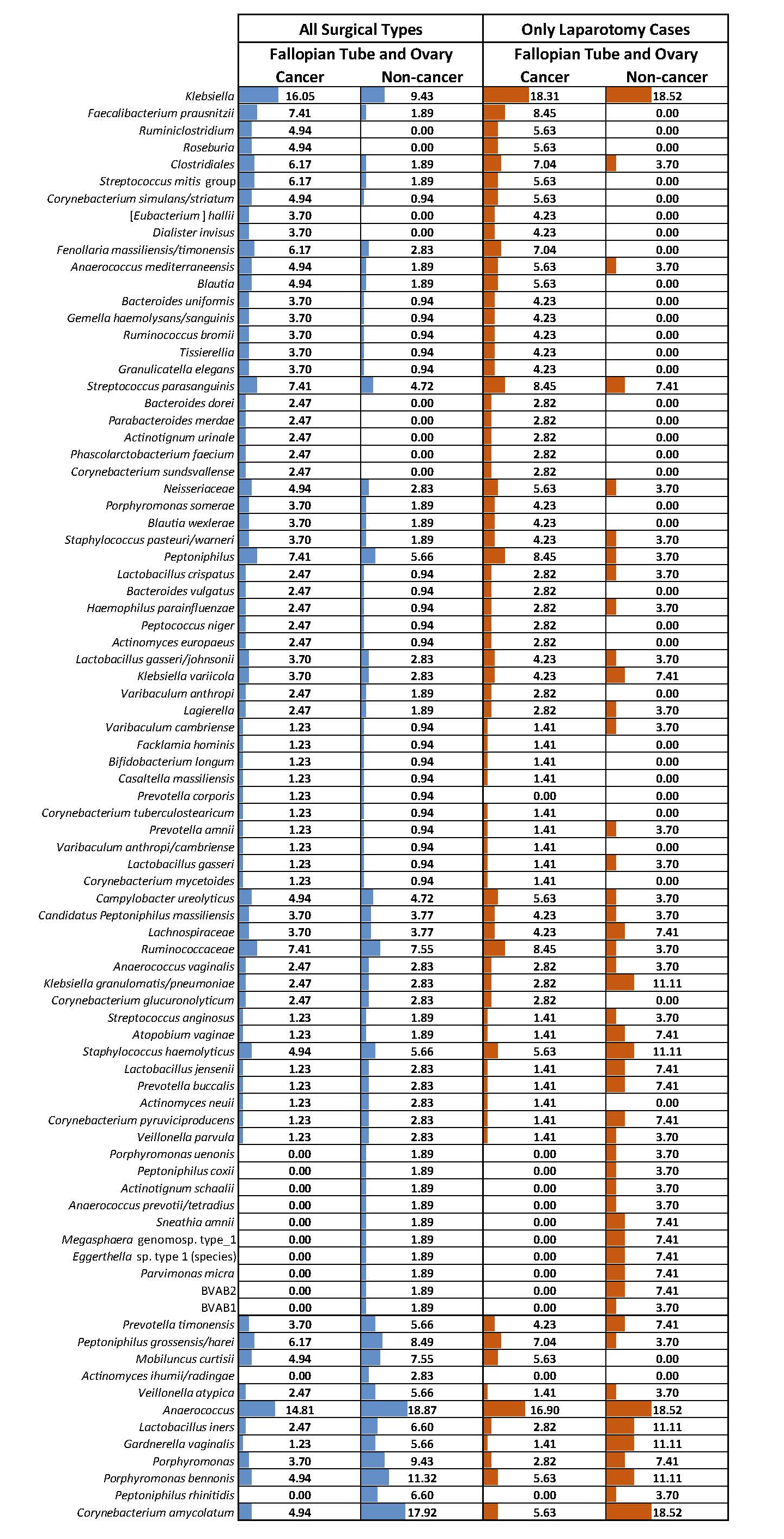
**
