# Supplementary material for: Identification of fallopian tube microbiota and its association with ovarian cancer
Source: eLife. 2024 Mar 7;12:RP89830. doi: 10.7554/eLife.89830 (PMC10942644; doi:10.7554/eLife.89830)
Supplement: Supplementary file 5. — Each number is the percentage of individuals in each category with the presence of each bacterial species. [file elife-89830-supp5.docx]

**Supplemental Table 5.** The bacterial prevalence in FT samples from non-cancer patients: comparison of laparoscopic/robotic and laparotomy cases. Each number is the percentage of individuals in each category with the presence of each bacterial species.

**
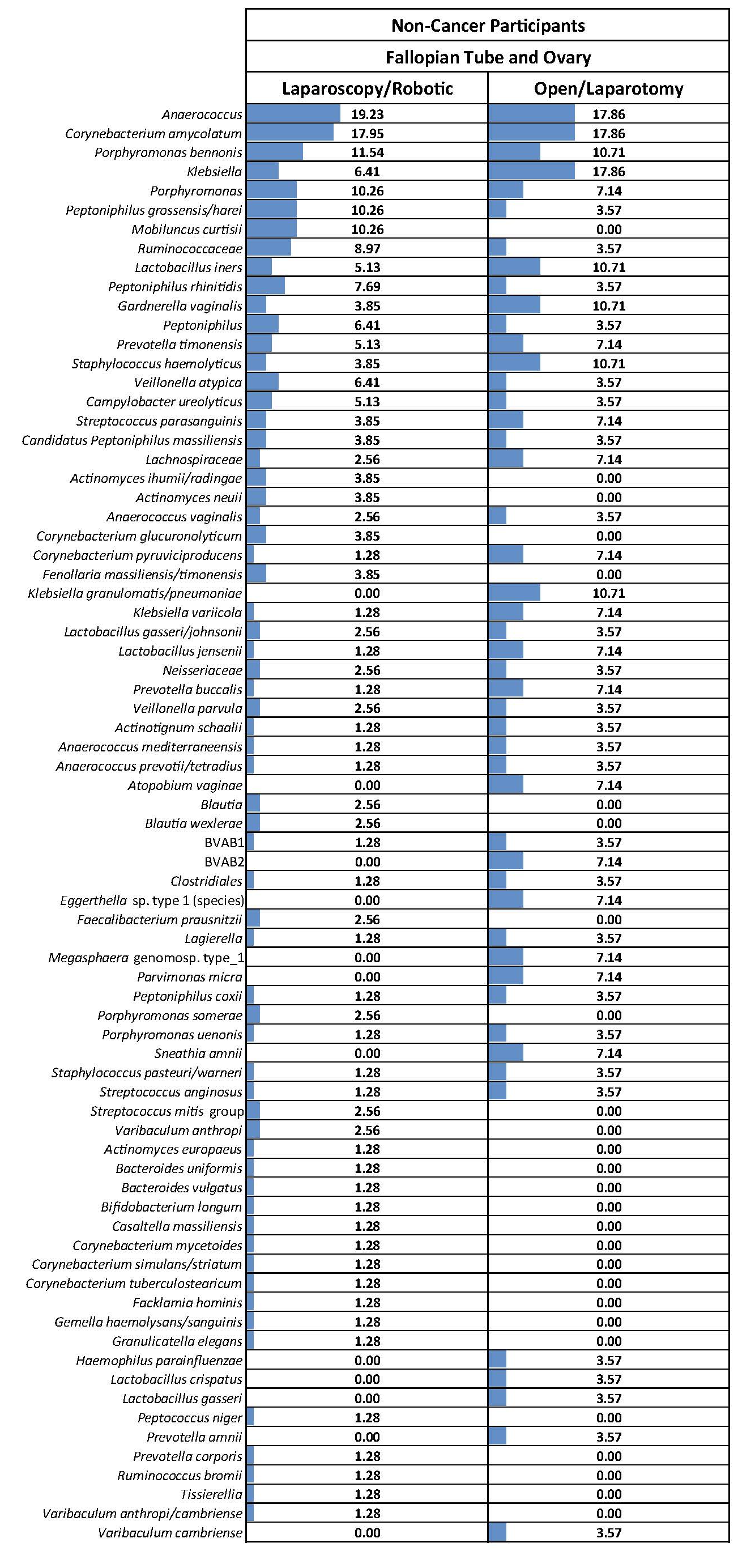
**
